# Action Enhances Acoustic Cues for 3-D Target Localization by Echolocating Bats

**DOI:** 10.1371/journal.pbio.1002544

**Published:** 2016-09-08

**Authors:** Melville J. Wohlgemuth, Ninad B. Kothari, Cynthia F. Moss

**Affiliations:** 1 Department of Psychology and Institute for Systems Research, Program in Neuroscience and Cognitive Science, University of Maryland, College Park, Maryland, United States of America; 2 Department of Psychological and Brain Sciences, Johns Hopkins University, Baltimore, Maryland, United States of America; Queen Mary University of London, UNITED KINGDOM

## Abstract

Under natural conditions, animals encounter a barrage of sensory information from which they must select and interpret biologically relevant signals. Active sensing can facilitate this process by engaging motor systems in the sampling of sensory information. The echolocating bat serves as an excellent model to investigate the coupling between action and sensing because it adaptively controls both the acoustic signals used to probe the environment and movements to receive echoes at the auditory periphery. We report here that the echolocating bat controls the features of its sonar vocalizations in tandem with the positioning of the outer ears to maximize acoustic cues for target detection and localization. The bat’s adaptive control of sonar vocalizations and ear positioning occurs on a millisecond timescale to capture spatial information from arriving echoes, as well as on a longer timescale to track target movement. Our results demonstrate that purposeful control over sonar sound production and reception can serve to improve acoustic cues for localization tasks. This finding also highlights the general importance of movement to sensory processing across animal species. Finally, our discoveries point to important parallels between spatial perception by echolocation and vision.

## Abstract 2

As an echolocating bat tracks a moving target, it produces head waggles and adjusts the separation of the tips of its ears to enhance cues for target detection and localization. These findings suggest parallels in active sensing between echolocation and vision.

## Author Summary

As animals operate in the natural environment, they must detect and process relevant sensory information embedded in complex and noisy signals. One strategy to overcome this challenge is to use active sensing or behavioral adjustments to extract sensory information from a selected region of the environment. We studied one of nature’s champions in auditory active sensing—the echolocating bat—to understand how this animal extracts task-relevant acoustic cues to detect and track a moving target. The bat produces high-frequency vocalizations and processes information carried by returning echoes to navigate and catch prey. This animal serves as an excellent model of active sensing because both sonar signal transmission and echo reception are under the animal’s active control. We used high-speed stereo video images of the bat’s head and ear movements, along with synchronized audio recordings, to study how the bat coordinates adaptive motor behaviors when detecting and tracking moving prey. We found that the bat synchronizes changes in sonar vocal production with changes in the movements of the head and ears to enhance acoustic cues for target detection and localization.

## Introduction

Animals experience complex and noisy sensory information when navigating in the natural environment, and they must employ strategies to selectively sample and process biologically relevant signals in dynamic contexts [1,2]. One strategy for parsing complex sensory information is “active sensing,” which engages motor systems in the acquisition of sensory information [2,3]. Behavioral active sensing, the focus of the research presented here, can take two forms: (1) the generation of signals which the animal uses to probe the environment and (2) the purposeful orienting of peripheral sensory organs towards a selected target [2–4]. In humans and other animals that rely heavily on vision, active sensing involves orienting of gaze toward an object of interest to gain higher resolution of the visual scene [2,3,5]. Humans also employ behavioral adjustments during auditory localization in the form of head movements [6,7], demonstrating that active sensing is a generalized strategy for gathering sensory information.

The echolocating bat presents a powerful model to investigate active sensing for auditory spatial localization and tracking, as this animal adaptively controls the generation and reception of the sensory signals it uses to represent the environment through adjustments to sonar call production and echo acquisition on a very rapid timescale [8,9]. It is well established that bats of different species adapt the features of echolocation calls as they detect, track, and intercept prey [9–11], but comparatively little is known about the active orienting of the head and ears to improve sonar target detection and localization [12,13]. Early research in the horseshoe bat—a species that emits long duration (~50–200 ms), constant frequency (CF) calls, in combination with brief frequency modulated (FM) sweeps—demonstrated that they time the sweeping movements of the pinna in the anterior/posterior direction with the arrival of an echo from a stationary target [14]. By moving the pinna during echo reception, the bat induces amplitude modulations and Doppler shifts in opposing directions at the two ears, amplifying inter-aural differences in the features of echoes [15,16].

The big brown bat (*Eptesicus fuscus*) emits short duration (0.5–15 ms), FM down-sweeps and successfully forages in both open and cluttered spaces and in the presence of nearby conspecifics [11,17,18]. These behaviors require fine control over sonar signal production and echo reception to extract biologically relevant signals from streams of acoustic stimuli. Here, we employed a novel method to measure in precise detail the dynamic coordination of sonar signal timing with head and ear movements as the bat tracked and intercepted prey from a platform. Our data demonstrate that the bat temporally synchronizes sonar vocalizations with changes in the 3-D orientation of the head and external ears to enhance cues for target detection and localization. Specifically, we discovered that the bat “waggles” the head to alter the relative elevation of the ears, while also changing the separation between the tips of the ears as it engages in target tracking. We hypothesize that the tight temporal coupling between adaptive sonar vocalizations and head/ear positioning is integral to high-resolution acoustic imaging by sonar and, more generally, applicable to sensory capture through active sensing. Our findings also reveal parallels between active vision and echolocation that point to general principles of spatial localization across sensory systems.

## Results

### Range-Dependent Adjustments in Sonar Vocalizations

**Fig 1:**
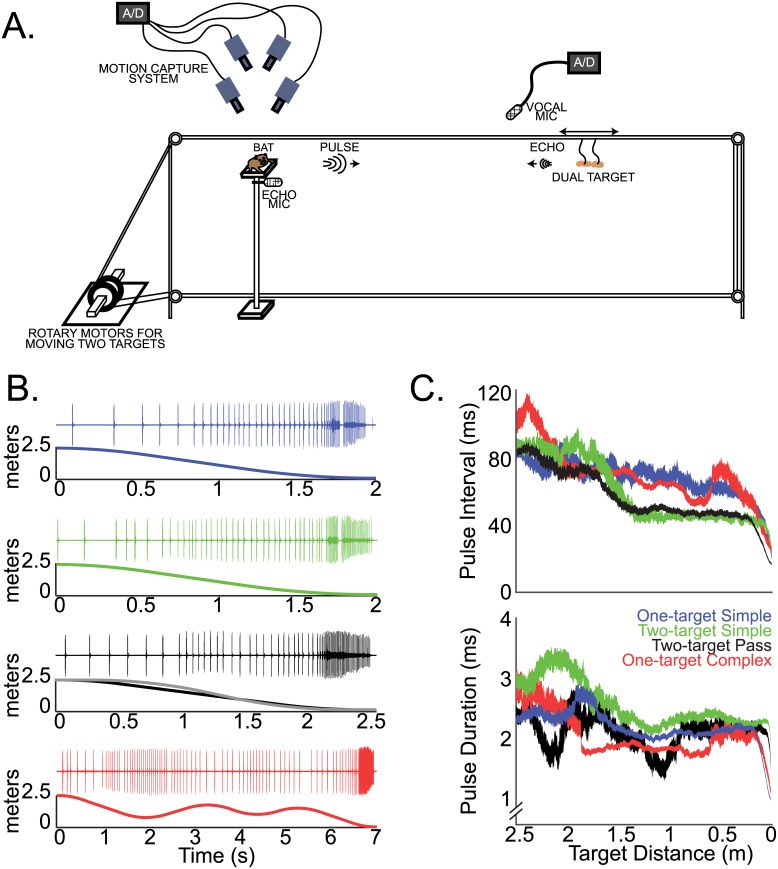
Behavioral setup and adaptive echolocation call changes. (A) Bats are trained to rest on a platform and track a moving target (tethered mealworm) by echolocation in the dark. The target(s) is moved via a rotary stepper motor attached to monofilament wire that is looped around a set of four pulleys. While the bat tracks the target(s), one microphone records the bat’s vocalizations, and a second microphone records the returning echoes. The 3-D positions of the head and pinna are recorded with a set of four infrared (IR) motion capture cameras. (B) Example sonar oscillograms (top) and target distance versus time plot (bottom) for each target motion condition presented to the bat (top to bottom): one-target-simple motion, two-target simple motion, two-target-pass motion, and one-target complex motion. (C) Top, mean +/- standard error of the mean (s.e.m) change in pulse interval over target distance for different varieties of target motion (colors as in B, significant differences are indicated in S1 Fig). Bottom, mean +/- s.e.m. change in pulse duration over target distance for each target motion condition (colors as in B, significant differences are indicated in S2 Fig). Data for this figure can be found at http://dx.doi.org/10.7281/T1W66HPZ.

Three big brown bats (*E*. *fuscus*) were trained to track moving prey items from a platform (experimental setup in Fig 1A and S1 Movie). By using this experimental paradigm, the position and movement of the insect target could be precisely controlled with respect to the bat, and detailed measurements could be made of the bat’s ear and head positions. Example oscillograms of sonar pulse sequences produced by the bat as it tracked targets with four distinct motion trajectories (see Table 1) are shown in Fig 1B (one-target simple, two-target simple, two-target pass, and one-target complex motions). One-target simple involves forward motion of a single tethered insect towards the bat’s position on the platform, and two-target simple involves the simultaneous forward motion of two insects on adjacent, parallel strings (Fig 1A). In the two-target pass condition, one slower moving target initially approaches the bat and a second target starts 0.5 s later, overtaking the first around 1 m in front of the animal. In the complex target motion condition, a single tethered insect approaches the bat and then recedes twice before finally moving forward to reach the bat. (see Table 1 for specific motion parameters). Note that each sonar vocal sequence exhibits a decreasing pulse interval with decreasing target distance and the stereotyped “buzz phase” just before target interception [8,17,19,20].

**Table 1:** Motion parameters and number of trials for each variety of target motion.

Movement Type	Starting Distance	Number of trials	Velocity	Motion Description
One-Target Simple	2.5 meters	98 trials	Target 1: 4 m/s	Towards the bat
Two-Target Simple	2.5 meters	36 trials	Target 1: 4 m/s Target 2: 4 m/s	Towards the bat
Two-Target Pass	2.5 meters	52 trials	Target 1: 3 m/s, Target 2: 6 m/s	Target 1 movement begins, followed by Target 2 (delayed by 0.5 seconds)
One-Target Complex	2.5 meters	42 trials	Target 1: 4 m/s	Direction changes at 0.75, 1.75, 1.0, and 1.5 meters

As reported for bats foraging on the wing [11], animals here exhibited adaptive modifications in sonar acoustic features with changing target distance (Fig 1C). It is also noteworthy that bats decreased pulse interval when tracking two targets, as compared with a single target (Fig 1C, top; S1 Fig). In addition to pulse interval, bats showed range-dependent adjustments in sonar pulse duration to avoid an overlap in time between sonar pulse emission and echo arrival (Fig 1C, bottom) [8,15]. Furthermore, the bats showed consistent modifications in pulse duration, which varied with target motion condition (Fig 1C, bottom; S2 Fig). Specifically, bats increased call duration when tracking two targets, as compared to one (S2B Fig). Longer call durations result in echo-echo overlap from the two adjacent targets, and previous research has shown that bats can resolve echo-echo overlap from spectro-temporal information contained in the composite sonar returns [21]. Bats also decreased call duration when tracking one-target complex motion, as compared to one-target simple motion (S2A Fig), demonstrating how the specific target motion trajectories can influence sonar vocal production.

In addition to adaptations of individual sonar pulses, it has been shown that bats modify the temporal dynamics of pulses into what has been termed “sonar sound groups” (e.g., reference [22]). Sonar sound groups are defined as clusters of two or more pulses separated by a short interval and flanked by pulses at longer intervals (S3A Fig). The incidence of sound group production varies with sonar task demands, and prior work demonstrated that bats increase the production of sonar sound groups when localization demands are high [22–26]. In agreement with these earlier results, we found that bats in the current study increased sonar sound group production during the more difficult tracking tasks of two-target-pass and one-target complex motion, as compared to one- and two-target-simple motion (S3B Fig, *t*-test, *p* < 0.0001, df = 118 for one-target complex/one-target simple; *t*-test, *p* < 0.0001, df = 161 for two-target pass/one-target simple). Additionally, as reported previously [22], bats in the current study produced sonar sound groups while tracking a target from a stationary position. This behavior demonstrates a decoupling between the coordination of sonar sound group production and the wing beat cycle of an echolocating bat (see, for example, references [27] and [28]).

### Head and Ear Movements

#### Head waggles

**Fig 2:**
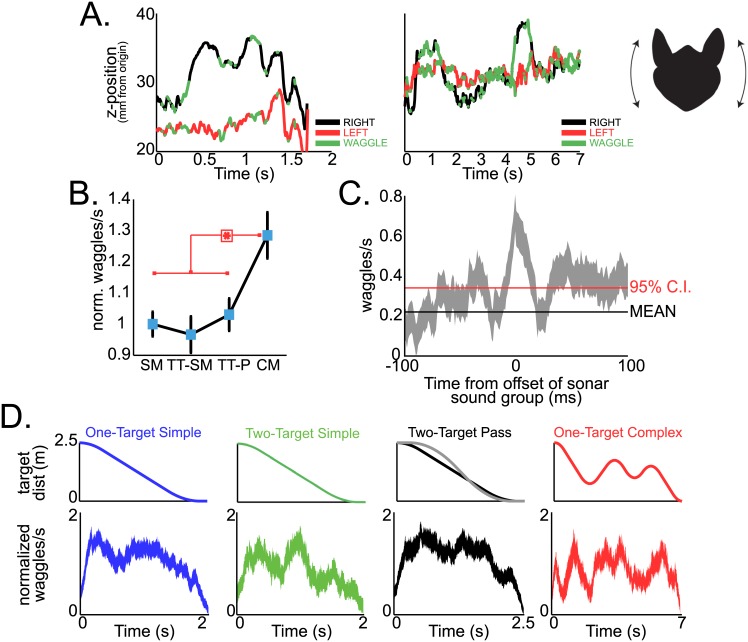
Head waggles and target motion. (A) Incidence of head waggle for Bat 1 during one-target simple (left) and one-target complex (right) motion. Displayed are the z-positions (height) of right ear (black) and left ear (red), with times of head waggles displayed in green. Notice the increase in head waggles for one-target complex motion. Far right panel displays a cartoon of the head waggle motion. (B) Summary across all bats for normalized head waggles per second for four different types of target motion. There is a significantly higher number of head waggles per second for one-target complex motions than all other types of target motion (permutation test, *p* < 0.0001). (C) Mean +/- s.e.m. of head waggles per second at the conclusion of a sonar sound group. The *zero* time point is the offset of the last sonar pulse in a sonar sound group. Plotted over these data are the mean waggles per second outside the context of sonar sound group production (black) and the 95% confidence interval on the mean in red. (D) Left to right, normalized head waggles per second for one-target simple, two-target simple, two-target pass, and one-target complex motions. The top row is the target position throughout trials; the bottom row is the target distance aligned normalized mean +/- s.e.m. of head waggles per second for each variety of target motion. Data for this figure can be found at http://dx.doi.org/10.7281/T1W66HPZ.

As bats tracked moving targets from the platform, they made distinctive head movements, which we refer to here as “head waggles.” Head waggles involve rotations of the head, resulting in changes in the relative elevation of the external ears (see Fig 2A, far right inset, and S2 Movie). Shown in Fig 2A are the vertical displacements of the right (black) and left (red) pinnae in elevation and the times when the two pinnae were moving in opposite directions (highlighted in green) in a bat tracking targets under two different motion conditions. We defined head waggles as events when the two pinnae were moving in opposite, vertical directions. The left panel of Fig 2A shows head waggle incidence in one trial when the bat tracked the tethered insect in the one-target simple motion condition; the right panel shows data from one trial of the one-target complex motion condition. These data illustrate that the bat increases the incidence of head waggles during more complex motion tracking (more green highlighted regions during one-target complex than one-target simple target tracking). Indeed, all bats studied here showed a significant increase in the number of head waggles in one-target complex motion trials over all other trial motion conditions (Fig 2B, right panel, permutation test, *p* < 0.001 for all significant differences), demonstrating that the bats actively produced head waggles when tracking erratically moving prey.

Bats in the current study coordinated head waggles with changes in sonar vocal production. Bats showed an increase in the number of head waggles after the conclusion of a sonar sound group, suggesting the bat’s dual control over the timing and reception of sonar signals during target tracking (Fig 2C). Specifically, our data show a significant increase in head waggles per second after sonar sound group production over times when the bat was not producing sonar sound groups (see Fig 2C, 95% confidence interval in red).

Additionally, bats performed head waggles at specific instances of target motion. First, bats increased head waggles across all target motion conditions when the target just began to approach the bat (Fig 2D). Bats in the current study also increased head waggles when the target abruptly changed directions, as seen in the data from one-target complex motion trials (Fig 2D, right panel). These data indicate that head waggles are used to support target localization and tracking.

In order to determine how head waggles alter the acoustic view of the bat, we calculated the vertical displacement in degrees between the two ears and the target. Changing the vertical positions of the ears alters inter-aural differences, which can in turn contribute to greater spatial localization accuracy [29]. We examined the vertical offset of the ears by measuring the maximum vertical displacement of the tips of the two pinnae and then calculating their angle with respect to the position of the target (S4 Fig). The analysis reveals the full range of angular offsets in vertical pinna position, including values up to four degrees (S4 Fig, inset). The maximum angular offset between the two ears occurred for target distances between 1 and 1.5 meters, and the average across all trails and conditions was 0.70 degrees.

#### Adaptive control of inter-pinna separation

**Fig 3:**
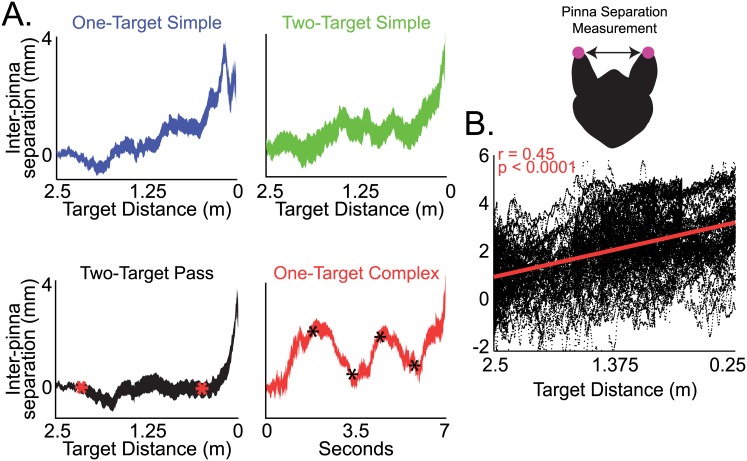
Changes in pinna separation with target distance for each target motion condition (colors as in Fig 1). (A) Top left, change in inter-pinna separation as a function of target distance for one-target simple motion. Plotted is the mean +/- s.e.m. for all trials. Values are normalized to zero for the starting position at the beginning of each trial. Top right, normalized data for two-target same motions; bottom left, normalized data for two-target pass motions (asterisks indicate when the second target begins to move, and when it overtakes the first target); bottom right, normalized data for one-target complex motions (asterisks indicate times when the target changes motion direction). (B) Normalized inter-pinna separation as a function of target distance for all varieties of target motion. There is a significant correlation between decreasing target distance and increasing pinna separation (Pearson’s correlation, r = 0.45, *p* < 0.0001). Inset details inter-pinna separation measurement. Data for this figure can be found at http://dx.doi.org/10.7281/T1W66HPZ.

In addition to measuring head movements and the relative elevation of the pinnae, we also investigated the motions of the pinnae that are independent of the movements of the head. Considering the importance of inter-aural cues for sound localization, we analyzed differences in inter-pinna separation across target motion conditions (see S3 Movie). Bats in the current study adjusted inter-pinna separation by either raising the tips of the pinnae to decrease the separation distance or by “flattening” the pinnae positions, i.e., lowering the tips of the pinnae and thereby increasing the separation distance (S5 Fig). Displayed in Fig 3A are the normalized changes in inter-pinna separation across all bats for each target motion condition. These data show that bats reliably increased inter-pinna separation as the target approached, and this occurred for all target motion conditions. Adaptive changes in inter-pinna separation with target distance are most apparent in one-target complex motion trials (Fig 3A, bottom right panel in red), in which inter-pinna separation oscillated in synchrony with the oscillations in target distance (asterisk indicates change in direction of target motion). The changes in the bat’s inter-pinna separation as it tracked an oscillating target showed a mean response time to a change in target direction of approximately 450 ms (S6 Fig). This is the time lag between the change in inter-pinna separation after the change in target motion direction.

Collapsing data from all bats and all target motion conditions reveals a significant, positive correlation between decreasing target distance and increasing inter-pinna separation (Fig 3B, Pearson’s correlation, r = 0.45, *p* < 0.0001). Although the overall change in inter-pinna separation with target distance can be described as a linear function when averaged over trials, on a trial to trial basis, inter-pinna separation showed some variability in its relationship to target distance (S7 Fig). We observed inter-pinna separation mostly changing gradually with target distance (S7 Fig), but there were also occasional, abrupt, small changes on a shorter timescale. This variability on a short timescale contributes to the scatter in Fig 3B.

**Fig 4:**
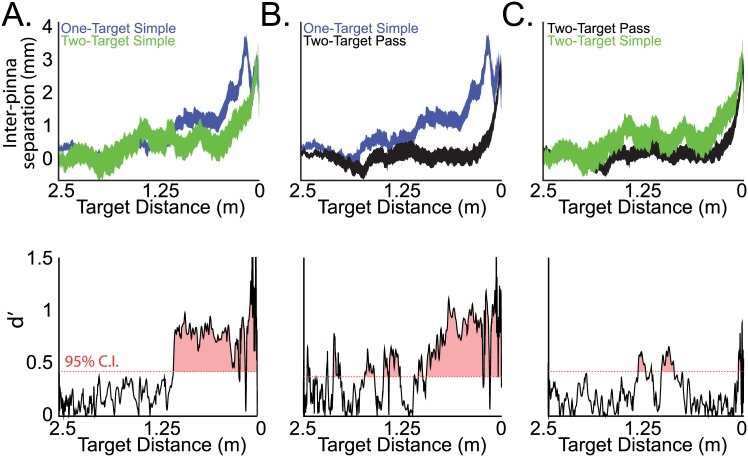
(A) Top, normalized inter-pinna separations for one-target simple motion (blue) versus two-target simple motions (green) combined across bats. Plotted are the mean +/- s.e.m. of the inter-pinna separation distances as a function of target distance. Bottom, dʹ calculation, or discriminability index, between one-target simple motion and two-target simple motion inter-pinna separation. Red shaded region indicates dʹ values above the 95% confidence interval as determined by a permutation test, indicating time points of significant differences in inter-pinna separation. (B) Top, normalized inter-pinna separations for one-target simple motion (blue) versus two-target pass motions (black) combined across bats. Bottom, dʹ calculation between one-target simple and two-target pass motion conditions. Red shaded region indicates significant differences. (C) Top, normalized inter-pinna separations for two-target simple motion (green) versus two-target pass motions (black) combined across bats. Bottom, dʹ calculation between two-target simple and two-target pass motion conditions. Red shaded region indicates significant differences. Data for this figure can be found at http://dx.doi.org/10.7281/T1W66HPZ.

Importantly, bats also adapted pinna position in response to the specific target motion parameters. Fig 4 plots the mean +/- s.e.m. of the normalized inter-pinna separations between different pairs of target motion conditions, and these differences were found to be statistically significant. Fig 4A (top panel) shows data for inter-pinna separation comparisons between one-target and two-target simple motions. Note that bats decreased inter-pinna separation when tracking multiple targets at shorter distances (non-overlapping regions in top panel starting at ~1-meter target distance; bottom panel, red-shaded region indicating dʹ values above 95% confidence interval). Inter-pinna separation was also significantly reduced for two-target pass motion, as compared to one-target simple motion (Fig 4B, top, non-overlapping region starting at ~1-meter target distances; bottom, red-shaded region indicating dʹ values above 95% confidence interval). Comparisons of inter-pinna separation for two-target simple and two-target pass motions revealed a small decrease in inter-pinna separation for two-target pass motions at intermediate distances and right before target capture (Fig 4C, top, non-overlapping regions; bottom, red-shaded region indicating dʹ values above 95% confidence interval).

To summarize, our data show that bats adapt inter-pinna separation with respect to target distance and across the target motion parameters. These results demonstrate the adaptive modifications in pinna position that serve to enhance echo cues for target detection and localization.

### Coordinating Signal Production and Reception

#### Inter-pinna separation and pulse/echo timing

**Fig 5:**
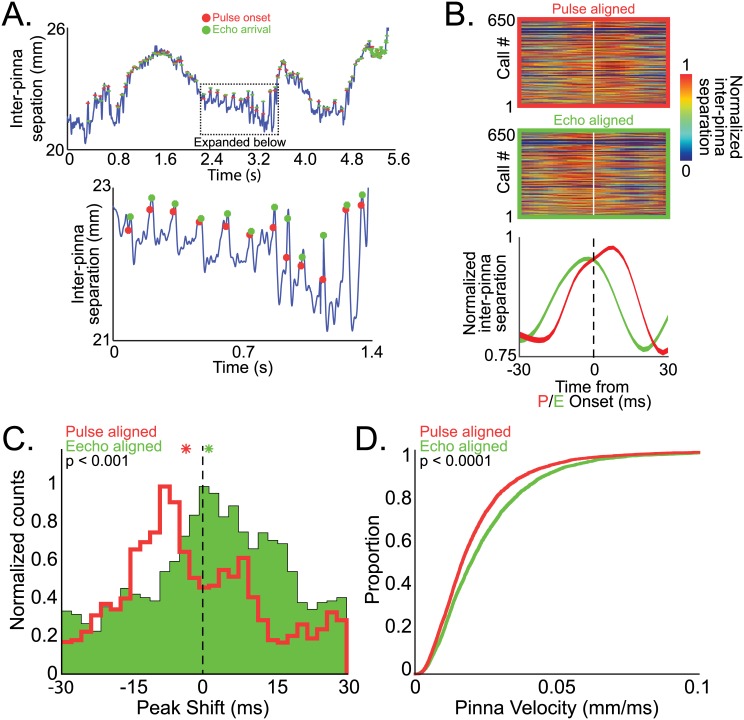
Local changes in inter-pinna separation are tied to sonar signals. (A) Example of changes in inner-pinna distance for a bat tracking a tethered insect in a one-target complex motion trial. On a global scale, the bat changes inter-pinna separation with changing target distance; locally, there is also a small increase in inter-pinna separation near vocal onset time. Red dots indicate time of sonar call emission; green dots indicate time of sonar echo arrival. (B) Top, normalized individual pulse aligned (top panel) and echo aligned (bottom panel) inter-pinna separations. Red indicates the largest separation between the pinna, blue the smallest, in a 60 ms window aligned to the time of pulse emission or echo arrival. Bottom, mean +/- s.e.m. of normalized local peaks in inter-pinna distance aligned to either the sonar pulse onset (red) or echo arrival time (green). (C) All shifts in the peak of inter-pinna separation aligned to pulse onset (red) and echo arrival (green). Asterisks indicate the average shift in the peak displacement of inter-pinna separation around the time of pulse emission/echo arrival. The two distributions are significantly different (permutation test, *p* < 0.001). (D) Velocity of pinna motion during pulse production (red) and echo arrival (green). Pinna velocity is significantly higher during echo reception than during pulse production (permutation test, *p* < 0.0001). Data for this figure can be found at http://dx.doi.org/10.7281/T1W66HPZ.

The bats produced small deflections in the pinnae with each sonar vocalization, which served to produce further increases in inter-pinna separation as the target approached. Shown in the top panel of Fig 5A is the change in inter-pinna separation for a bat tracking a target during a one-target complex motion trial. Global changes in inter-pinna separation are apparent as the target moved back and forth, but, in addition, the bat also made small, rapid deflections in inter-pinna separation around the time of each sonar call emission. This is shown more clearly in the time-expanded portion presented in the bottom panel of Fig 5A. Sonar vocalization onset times are indicated in red, and echo arrival times are indicated in green. This figure illustrates that when the bat vocalizes, there is often a time-locked increase in inter-pinna separation. We call these pinna deflections “local” peaks in inter-pinna separation.

We examined the timing of the local peaks in inter-pinna separation with respect to sonar pulse onset and echo arrival time to determine whether the pulse emission or echo return was more closely timed with the peaks in local pinna deflections. In the top two panels of Fig 5B are heat plots of normalized local peaks in inter-pinna separations time aligned to either sonar pulse onset (red, top panel, white line indicates pulse onset) or echo arrival (green, middle panel, white line indicates echo arrival time). The bottom panel of Fig 5B displays the mean +/- s.e.m. normalized movements of the pinnae aligned to pulse onset (red) or echo arrival (green). These data demonstrate that echo arrival time is more closely aligned with the local peak in inter-pinna separation than sonar vocalization onset time (i.e., better peak alignment for echo arrival time than sonar onset time). We calculated the time shift between the local peak in the inter-pinna separation and sonar pulse onset time (Fig 5C, red) and echo arrival time (Fig 5C, green) to quantify the delay between pulse/echo time and the inter-pinna separation peak (zero indicates that pulse/echo time occurs at the exact time of the local peak in inter-pinna separation). The average time delay between the local peaks in pinna separation and echo arrival fell close to zero-lag, indicating an almost synchronous timing of pinna deflection and echo arrival. The two distributions are significantly different (permutation test, *p* < 0.001)

To determine if these small oscillatory events around the time of sonar vocalizations were the indirect result of the bat opening its mouth, or if the bat can decouple movements of the pinnae from opening of the mouth, we examined inter-pinna separations at times when the bat was not vocalizing, as well as times when the bat vocalized without a concomitant change in pinna position. Our rationale was that if the bat produced small oscillatory pinna movements when it was not vocalizing, or if the animal vocalized without a change in pinna position, then the two behaviors are not necessarily directly coupled. We do indeed find many instances when the bat made small deflections in pinna position when it was not vocalizing (S8 Fig, blue arrows). In addition, we also observe times when multiple vocalizations were produced during one oscillatory cycle of the local peaks in inter-pinna separation (S8 Fig, green arrows). And finally, we find instances when the animal vocalized without a local peak in inter-pinna separation (S8 Fig, red arrows). These data demonstrate that the bat can make changes in the movements of the pinna that are independent of its vocal behaviors, providing evidence that these movements are not always directly coupled.

Lastly, considering earlier work showing how dynamic movements may contribute to auditory localization, we further characterized the pinnae movements during echo reception. To do so, we compared the velocity of the pinnae during sonar vocal production to the velocity of the pinnae during echo reception, assuming that the echo duration was the same as sonar pulse duration. Interestingly, the velocity of pinna movements was also higher during echo arrival than sonar pulse production (Fig 5D, permutation test, *p* < 0.0001). These data suggest another way in which the bat’s orienting movements can modify reception of acoustic signals.

## Discussion

Using a novel method to investigate adaptive motor behaviors of echolocating bats engaged in target tracking, we report here that animals rapidly and tightly coordinated sonar signal timing with ear and head movements in relation to target distance and motion trajectory. Bats also showed adjustments in sonar pulse duration and interval, as well as the production of sonar sound groups, with the distance and trajectory of the target. The bat’s adaptive echolocation behavior was accompanied by rapid changes in head orientation and external ear (pinna) position, which collectively influence the acoustic cues used to localize and track the moving target(s) [9,11]. These findings demonstrate that auditory feedback in the echolocating bat drives very rapid motor adjustments in both sonar call production and head/ear movements on a millisecond timescale.

In the current study, bats increased sonar pulse rate and call duration when tracking two targets compared to a single target. Furthermore, bats increased the production of sonar sound groups while tracking the target in the complex motion condition, an adaptive echolocation behavior that is hypothesized to increase sonar localization accuracy [9,11,22–26]. Through these adjustments in sonar vocal production, the bat directly controls the sensory information available to the auditory system during target localization and tracking.

Importantly, bats also produced head waggles after the production of sonar sound groups. It has been shown in humans that movements of the head and body, active or induced, result in significantly increased acuity of sound localization [30–32]. Head waggles in the bat change the relative elevation of the two external ears, providing changes in the binaural cues carried by echo streams [33]. Specifically, the head waggles serve to modify and enhance interaural level difference (ILD) changes, as well as inter-aural spectral differences, of successively returning echoes from a sonar sound group. Indeed, the angular offset of the pinnae during head waggles alters the vertical positions of the external ear by up to four degrees. Previous head-related transfer function (HRTF) measurements in the big brown bat report a prominent spectral notch that decreases in frequency with sound source elevation [34,35]. In these studies, the spectral notch shifts by approximately 5 kHz for each 5-degree step in sound source elevation around the horizon, providing an indication of large differences between power spectra of echoes received at the two ears during a head waggle.

Previous research in humans report that “rhythmic sampling” of the environment increases acoustic resolution by allowing the brain to anticipate and prepare for future sensory updates [6,7]. The echolocating bat’s production of sonar sound groups (sonar sounds with regular intervals) results in cascades of echoes returning at regular intervals [22], potentially allowing the bat to anticipate the temporal patterning of echo returns. Timing head waggles across a series of echo returns could provide the bat with contrasting echo information, improving the distance and direction localization of a moving target in a manner similar to motion parallax in visually guided animals [36]. For visual motion parallax, small movements of the eyes have been found to accentuate depth perception and the tuning curves of neurons in visual area 5, also known as middle temporal (MT) [37]. Using head waggles, the bats can similarly induce a related perceptual phenomenon via acoustic parallax. Acoustic parallax has been hypothesized to play a role in distance perception of auditory objects: a near auditory object moving across the horizon has a higher rate of ILD changes than a far object [38,39]. In this way, auditory objects at different ranges are tagged with specific changes in ILD over time. It has been reported that head movements by human listeners similarly assist in the segregation of multiple auditory streams by tagging each stream with specific inter-aural differences [40], and that these movements contribute to distance perception [41]. We propose that the coupling sonar sound groups with head waggles serves to increase auditory localization accuracy in bats, much in the same way as rhythmic sampling and motor-induced ILD change hone auditory processing in humans.

Echolocating bats tracking targets in the current study also adjusted the pinna position with respect to the head by altering the separation between the tips of the outer ears. These adjustments varied with target distance, changes in target trajectory, and the number of targets being tracked. In general, as a target approached, the separation between the bat’s pinnae significantly increased. We hypothesize that control over pinna separation aids in both target detection and localization. At greater target distances, smaller inter-pinna separations may aid in the collection of weak echo returns. Previous work on the greater horseshoe bat, a species that uses long duration, constant frequency (CF) sonar signals, suggests that a more erect pinna position (decreased inter-pinna separation), increases the bat’s sensitivity to echoes returning along the midline, whereas lowered pinna tips (increased separation) boost sensitivity at more peripheral locations [12]. Big brown bats that use frequency modulated (FM) signals may similarly enhance echo detection by directing the pinnae at the higher signal to noise ratio (SNR) components of echoes along the midline when target distance is larger [42]. At close target range, however, the bat’s acoustic field of view must be broad in order to ensure that the target remains in its spatial processing window, and increased inter-pinna separation serves to increase the bat’s acoustic field of view.

Increasing the separation and flattening the pinna at shorter target distances also supports sonar localization by augmenting ILD and interaural time difference (ITD) cues [12,43]. Although the small size of the bat’s head limits its range of ITD, sound level differences at the ears effectively boost inter-aural time differences through time-intensity trading [43]. By flattening the pinna positions, bats alter ILD values in ways that could further enhance echo localization.

In addition to the effects of target distance on inter-pinna separation, we observed that bats alter inter-pinna separation when tracking multiple targets. Bats in the current study decreased inter-pinna separation when tracking two targets as compared to one. Holding the pinna at a more upright and narrow orientation may also aid in the segregation of echoes from multiple targets by limiting the acoustic field of view to a more restricted azimuthal angle [12].

By altering the inter-pinna separation with target distance, the bats ultimately influence the acoustic information used to represent their acoustic scene [12]. Bats also dynamically modulate the sampling volume of their acoustic scene by altering the spectral content of their vocalizations [44]. Lower-frequency sounds are less directional than higher-frequency sounds, and when bats shift the frequencies of their vocalizations, they can increase or decrease their acoustic field of view [44]. Previous research has shown that bats typically downshift the frequency band of their vocalizations in the terminal buzz phase, an adjustment that increases their sonar field of view in the final attack phase of insect pursuit [45]. In our study, bats showed a similar downshift in the frequency of vocalizations with decreasing target distance (S9 Fig) while simultaneously increasing inter-pinna separation. By performing these coordinated behaviors, bats can correlate the width of the sonar beam with the width of their acoustic sensing. At larger target distances, the bats emit high-frequency, directional vocalizations while keeping inter-pinna separation narrow to focus hearing sensitivity along the midline. As the target approaches, sonar call frequency decreases and the sonar beam widens, while an increase in inter-pinna separation increases sensitivity to more peripheral locations. In this way, the acoustic view during echolocation is determined by both the change in the outgoing signal as well as a change in the positions of the external ears, and bats modify these parameters to meet specific task demands.

It is noteworthy that big brown bats rapidly coordinated small deflections of the pinnae around the time of sonar vocalizations and echo arrival. Previous research has reported this behavior in CF bats echolocating a stationary target [14,16,46]. These bats produce long sonar vocalizations and enhance inter-aural differences by coordinated pinna movements, which serve to increase localization accuracy [13]. Our work extends this finding to bats that use very brief FM sonar signals (0.5–4 ms in this study) to track a moving target. When tracking a moving target, the bat must continuously update information about target position from dynamic echo returns [9,47], and our data suggest that motor programs for vocal production and pinna deflections are tightly coordinated to support high-resolution sonar localization. Through the synchronized timing of sonar vocalizations and pinna/head movements, the bat influences the cues for sonar localization to enable a highly precise and flexible distal sensing system.

By quantifying the bat’s natural orienting behaviors in a novel target-tracking task, we discovered that echolocation shares with both human vision and audition general solutions to object localization in 3-D space. Much in the same way that humans move their eyes to foveate an object or move their heads to accentuate information about the position of an auditory or visual object, the bat controls sensory sampling through motor actions. The results of this study point to the importance of coordinated timing in active sensing systems that contribute to high-resolution spatial localization. The findings reported here not only hold importance to the field of systems neuroscience but also for engineering applications that implement adaptive controls for robotic sensory systems.

## Materials and Methods

### Behavioral Training

Three wild-caught big brown bats (*E*. *fuscus*) served as subjects in the following behavioral studies. The bats were collected in the state of Maryland under a permit issued by the Department of Natural Resources and were housed in an animal vivarium at the University of Maryland, College Park. All procedures employed were approved by the University of Maryland’s Institutional Animal Care and Use Committee (IRBnet Protocol 413006–4; UMD Protocol R-13-04).

The bats were trained to rest on a platform and track a moving, tethered prey item (mealworm) using echolocation (Fig 1A). The tethered prey item was suspended from a four-cornered loop of monofilament line that was connected to a set of four pulleys and a rotary stepper motor (Aerotech). The velocity, acceleration, and deceleration parameters could be set independently and were controlled via a computer interface with custom software written in Matlab 2012b. By spinning the rotary motors, the target could be moved in multiple directions, velocities, and accelerations in front of the bat.

Bats were initially trained by pairing a sound presentation with the delivery of a food reward immediately in front of the animal. Once the bat learned the association between the sound and food presentation, the starting position of the food reward, the time delay of its delivery, and the velocity of its travel were slowly increased as the animals learned to track the tethered target. The velocity of the target was steadily increased until it moved at 4 m/s, a velocity similar to the speed of a flying bat intercepting prey on the wing [48]. The final starting position of the target was 2.5 meters from the animal’s resting position on the platform. Catch trials were introduced to keep the bat actively engaged in the target tracking task rather than passively listening to sounds of the motors driving the target towards the bat. The catch trials involved suspending the target from a location adjacent to the target starting position on the loop of fishing line. The experimental apparatus therefore made the same sounds when propelling the target, but these sounds were decoupled from the target motion by having the target remain stationary at the starting position throughout the trial. Once the bat was trained on the task, approximately 25% of all trials were catch trials. Data collection began after animals exhibited reliable changes in pulse rate, pulse duration, and the production of the terminal “buzz phase” as the target approached.

### Target Trajectories

During the training phase, the target moved in one direction towards the animal at a velocity of 4 m/s. Once the bat reliably tracked the approaching target, more complicated target motions were presented to the animal, along with the addition of a second moving food reward. In total, four different types of target motion were presented to the animal (Fig 1B, Table 1), along with catch trials.

### Data Acquisition

The bat’s echolocation behavior, head aim, and pinnae movements were recorded as the animal tracked the moving tethered target. Time synchrony across video, audio, and other hardware devices was achieved through the generation of a TTL pulse by the computer controlling the stepper motor system. Data acquisition was collected on all systems starting with the TTL pulse at the beginning of target motion, and then continued for 10 s. This provided enough time for all the different target motion conditions to be completed with a buffer of at least 2 s.

Sonar vocalizations were recorded with an ultrasonic microphone manufactured by Ultra Sound Advice (SM2) connected to a pre-amplifier (SP2). The microphone was placed directly above the path of target motion and at a distance of 2.5 meters from the bat. The vocalizations were filtered between 15 and 110 kHz (Kemo VBF44) before being digitized at 250 kHz by a National Instruments M-series data acquisition board and saved on a hard drive for offline analyses.

Head and pinnae positions were tracked using a Vicon Nexus Motion Tracking System. Four T-40s series cameras were mounted above the bat on a custom built railing system. The cameras each had 18 mm lenses and were positioned to maximize overlap of each camera’s view of the platform area where the bat was tracking the target. The frame rate was set to 500 Hz and calibrated to sub-millimeter accuracy in XYZ position measurements with a moving wand-based calibration method. Three-millimeter diameter IR reflective markers were then affixed to the bat’s ears and nose-bridge using spirit gum. The positions of the reflective markers were tracked across the 2-D views of each Vicon camera and reconstructed in 3-D by the Vicon Nexus software (version 1.85). The x-dimension was set as left and right motion on the platform (perpendicular to target motion), the y-dimension was set as front to back motion, and the z-dimension was set as vertical motion. In summary, the bat’s vocalizations were recorded at a sampling rate of 250 kHz; the target’s position was measured at mm/ms; and the 3-D position of the head and ears was measured at 2 ms intervals and interpolated linearly to 1 ms intervals at a sub-millimeter spatial resolution.

### Data Analysis

Analysis of the 3-D positions of the head and pinnae was pre-processed using the Vicon Nexus platform. The positions of the left ear, right ear, and head markers were labeled in all frames where they were visible. Once the individual files were labeled, they were imported into Matlab 2012b. Matlab was first used to spline fill any gaps in the labeled marker trajectories. Gaps longer than 50 ms were not interpolated and were excluded from further analyses. Once the marker trajectories were post-processed, the Euclidian distances in three dimensions between the two tips of the outer ear pinnae markers and the rotational movements of the head were calculated. The inter-pinna separation over time was computed by measuring the absolute distance in 3-D space between each tip of the two pinnae. The rotational movement of the head was measured for periods of time when one pinna was moving down in the z-dimension while the other ear pinna was moving in an upwards direction for at least 20 ms. We term these asymmetrical rotational movements “head waggles,” and they were further confirmed by measurements showing that each pinna was also moving in opposite directions with respect to the head marker.

The analysis of the sonar vocalizations involved several steps, all of which were performed in Matlab 2012b. We first determined the onsets and offsets of each vocalization by drawing an amplitude threshold through a low-passed, rectified version of the original oscillogram. The onsets and offsets were then corrected for the travel time from the bat to the microphone (2.5 meters in front of the bat). Sonar pulse interval was calculated by taking the difference in time between onsets of successive sonar vocalizations, and sonar pulse duration was defined as the time between onset and offset time of each pulse. Echo arrival time was calculated by determining the distance of the target at the onset of each sonar vocalization and then using the speed of sound to estimate the travel time from the bat’s mouth to the target and then back to the bat’s ear. On each day, the temperature and humidity of the experimental room was recorded to accurately determine the speed of sound calculation. An ultrasonic microphone was also placed below the bat to record returning sonar echoes. The sensitivity of this microphone precluded recordings of sonar echoes returning from targets at larger distance, however. As a result, all echo arrival times were calculated as described above, and then these times were validated by examining when an echo was received on the microphone placed under the bat.

Once the times of the vocalizations were computed, the vocal sequences were analyzed to extract “sonar sound groups.” Sonar sound groups were determined by a statistical criterion defined in earlier work [9,22]. Briefly, sonar sound groups of three or more vocalizations were identified by a consistent pulse interval across at least three sonar vocalizations (within 5% error of the mean PI of the sound group), with the pulse interval of the surrounding calls at least 1.2 times larger than the mean sonar sound group pulse interval. For sonar sound groups of two vocalizations, the pulse interval of the flanking calls of the doublet sonar sound group had to be 1.2 times longer than the pulse interval between the two calls within the sonar sound group. In this way, the term “sonar sound group” identifies vocalizations that are in relative temporal isolation, and at shorter pulse intervals, from the surrounding sonar vocalizations.

The data on target motion, pulse timing, echo arrival, and sonar sound group production were then analyzed with respect to the pinnae and head movement data. The distance between the pinnae was measured as the bat tracked the moving target; the rotation of the head was analyzed with respect to target motion and sonar sound group production; and the timing of each vocalization and echo return was correlated with the timing of small pinna deflections. These small pinna movements are distinct from the global changes in inter-pinna distance that occur as the target approached. “Local peaks” in pinna separation were not observed when the target was in close proximity to the bat and sonar pulse rate was greater than 25 Hz, so this data was not included in subsequent analyses.

In order to calculate statistically significant differences between pulse interval and pulse duration across target motions, a dʹ statistic was calculated across target distance for all pair-wise comparisons of target motion. The dʹ statistic is a measure of discriminability between two time-varying signals. A random sampling method was then used to construct 95% confidence intervals (CI) where data from each group were pooled, randomly sampled into two groups, and then a dʹ statistic computed from the random permutation. This was repeated 1,000 times, and a distribution of randomly sampled dʹ statistics was constructed to determine the 95% CI. Pulse interval and pulse duration values were binned into 10 cm bins with respect to target distance.

A dʹ statistic was also used to determine time points of significant differences in inter-pinna separation for different motion conditions. As noted above, the dʹ statistic is a measure of discriminability and, in this case, the inter-pinna separation measurements. In order to determine 95% CI intervals, or when two data sets were significantly different, data were pooled and randomly shuffled into two groups, and a dʹ statistic was calculated across the randomly shuffled groups. This was performed 1,000 times, resulting in a distribution of dʹ values calculated across the randomly shuffled groups. The 95% confidence intervals on the dʹ statistic were determined from this distribution. For any instance of multiple comparisons, a Bonferroni correction was performed to determine the appropriate *p*-value.

## Supporting Information

S1 Fig Significant differences in sonar pulse interval across target motions. (A) D-prime (dʹ) calculation between one-target simple and one-target complex pulse intervals as a function of target distance. Red shaded regions indicate significant differences as determined by a permutation test, indicating time points of significant differences. (B) dʹ calculation between one-target simple and two-target simple pulse intervals as a function of target distance. (C) dʹ calculation between one-target simple and two-target pass pulse intervals as a function of target distance. (D) dʹ calculation between one-target complex and two-target simple pulse intervals as a function of target distance. (E) dʹ calculation between one-target complex and two-target pass pulse intervals as a function of target distance. (F) dʹ calculation between two-target simple and two-target pass pulse intervals as a function of target distance. Data for this figure can be found at http://dx.doi.org/10.7281/T1W66HPZ.
(EPS)

S2 Fig Significant differences in sonar pulse duration across target motions. (A) dʹ calculation between one-target simple and one-target complex pulse durations as a function of target distance. Red shaded regions indicate significant differences as determined by a permutation test, indicating time points of significant differences. (B) dʹ calculation between one-target simple and two-target simple pulse durations as a function of target distance. (C) dʹ calculation between one-target simple and two-target pass pulse durations as a function of target distance. (D) dʹ calculation between one-target complex and two-target simple pulse durations as a function of target distance. (E) dʹ calculation between one-target complex and two-target pass pulse durations as a function of target distance. (F) dʹ calculation between two-target simple and two-target pass pulse durations as a function of target distance. Data for this figure can be found at http://dx.doi.org/10.7281/T1W66HPZ.
(EPS)

S3 Fig Sonar sound group production. (A) Example of sonar sound group. Top, oscillogram of series of vocalizations produced while tracking a moving target. Bottom, highlight of three sonar sound groups: two doublets (two calls) and one triplet (three calls), demonstrating the shorter and regular pulse interval of the sonar group, surrounded by calls of longer pulse intervals. (B) Proportion of sonar sound groups per sonar pulse for each variety of target motion (left to right: one-target simple, two-target simple, two-target pass, one-target complex). There is a significant increase in sonar sound group production for two-target pass and one-target complex motions as compared to one-target simple motions (*t*-test, *p* < 0.0001 for all significant differences; df = 118 for one-target simple/one-target complex; df = 161 for two-target pass/one-target simple). Data for this figure can be found at http://dx.doi.org/10.7281/T1W66HPZ.
(EPS)

S4 Fig Degree offset of pinna during head waggles. Plotted is the maximum angular displacement of the tips of the pinnae during head waggles as a function of target distance. In black is the average maximum displacement, and the red shaded region indicates +/- 1 standard deviation of the average. Inset, distribution of all angles subtended by the two ears and the target in the vertical plane. Data for this figure can be found at http://dx.doi.org/10.7281/T1W66HPZ.
(EPS)

S5 Fig Raising and lowering the pinnae to change inter-pinna separation. Top, inter-pinna separation as measured by the distance between the tips of the pinna in three dimensions. Bottom, difference in elevation (*z*-axis) of tips of pinna and marker on the head. As inter-pinna distance decreases, there is a concomitant increase in the z-distance between the head and the pinna tip, indicating that the change in inter-pinna separation is related to either a lifting (decreased separation) or flattening (increased separation) of the pinna with respect to the head. Data for this figure can be found at http://dx.doi.org/10.7281/T1W66HPZ.
(EPS)

S6 Fig Time lag from change in target motion and change in pinna separation. (A) Top, one-target complex motion; bottom, inter-pinna separation for one trial of one-target complex motion. (B) Sliding cross correlations for ten example trials of inter-pinna separation during one-target complex tracking. Note that target motion was inverted in panel A for this analysis to maintain alignment of negative and positive slopes in the two traces for the cross correlation. The peak lag from zero of the cross correlation was chosen as the time lag between oscillations of target range and inter-pinna separation. Top inset, zoom of highlighted region; bottom inset, average time lag between the change in target motion and change in inter-pinna separation for 30 randomly selected trials (mean = -0.45 s delay). Data for this figure can be found at http://dx.doi.org/10.7281/T1W66HPZ.
(EPS)

S7 Fig Change in inter-pinna separation on a per-trial basis. Three individual trials demonstrating both the gradual change in inter-pinna separation with changing target distance, as well as abrupt, small changes on a shorter timescale. Data for this figure can be found at http://dx.doi.org/10.7281/T1W66HPZ.
(EPS)

S8 Fig Decoupling local peaks in inter-pinna separation with vocal behaviors. Shown are nine different trials of inter-pinna separation during the tracking of the one-target simple motion condition. Highlighted are three different instances of the decoupling pinna movements from sonar vocalizations. Blue arrows indicate times when local peaks in inter-pinna separation were performed without the synchronized production of a sonar vocalization. Green arrows indicate local peaks in inter-pinna separation where multiple vocalizations were produced during one oscillatory event of the pinna. Lastly, red arrows indicate times when the animal vocalized with no observable change in inter-pinna separation. Data for this figure can be found at http://dx.doi.org/10.7281/T1W66HPZ.
(EPS)

S9 Fig Change in sonar acoustics with target distance. Change in the end frequency of sonar vocalizations with decreasing target distance. Plotted is the mean +/- s.e.m., with end frequency significantly correlated with target distance (Pearson’s correlation, r = 0.34, *p* < 0.0001). Data for this figure can be found at http://dx.doi.org/10.7281/T1W66HPZ.
(EPS)

S1 Movie Tracking a moving target from a stationary position. In this three-part video, the bat is shown tracking a moving insect from a stationary position on a platform. The first segment is one trial of the bat tracking an insect from a view 3 meters in front of the bat. The second segment is a zoomed version of one trial of a bat tracking an insect. In both of these segments, the sounds that are heard are produced by a bat detector that makes the vocalizations of a bat audible to the human ear. The last segment was captured with high-speed video recording and has been slowed down by a factor of ten. Displayed on the bottom is the ongoing spectrographic representation of the bat’s vocalizations. The bat’s vocalizations are audible because the audio has been slowed down to match the recorded video.
(MP4)

S2 Movie Demonstration of the bats’ head waggle. This is a two-part movie showing the waggling of the head performed by the bat while tracking an insect. The first segment is shown at actual speed, and the bat’s vocalizations are audible through a bat detector. The second segment of the video was collected with high-speed recording and slowed down by a factor of ten, and again shows the distinctive head waggles performed by a bat while tracking an insect.
(MP4)

S3 Movie Measuring movements of the head and ears. This video has four different panels describing the technique for recording the 3-D positions of the head and ears in time synchrony with recordings of sonar vocalizations. The top-left panel displays the inter-pinna distance; the top-right panel displays the 3-D positions of the head and two ear markers; the bottom-left panel is a spectrographic display of the bat’s sonar vocalizations; and the bottom-right panel is a high-speed video recording of the bat while tracking a trial of the two-target pass motion condition.
(MP4)

## Abbreviations

CF: constant frequency
FM: frequency modulated
HRTF: head-related transfer function
ILD: interaural level difference
IR: infrared
ITD: interaural time difference
MT: middle temporal visual area 5
SNR: signal to noise ratio
